# Clinical results of dynamic tumor tracking intensity-modulated radiotherapy with real-time monitoring for pancreatic cancers using a gimbal mounted linac

**DOI:** 10.18632/oncotarget.25310

**Published:** 2018-05-04

**Authors:** Yoko Goto, Ryo Ashida, Akira Nakamura, Satoshi Itasaka, Keiko Shibuya, Mami Akimoto, Nobutaka Mukumoto, Shigemi Matsumoto, Masashi Kanai, Hiroyoshi Isoda, Toshihiko Masui, Yuzo Kodama, Mitsuhiro Nakamura, Kyoichi Takaori, Takashi Mizowaki, Masahiro Hiraoka

**Affiliations:** ^1^ Department of Radiation Oncology and Image-applied Therapy, Graduate School of Medicine, Kyoto University, Kyoto, Japan; ^2^ Department of Radiation Oncology, Graduate School of Medicine, Yamaguchi University, Yamaguchi, Japan; ^3^ Department of Clinical Oncology, Graduate School of Medicine, Kyoto University, Kyoto, Japan; ^4^ Department of Diagnostic Imaging and Nuclear Medicine, Graduate School of Medicine, Kyoto University, Kyoto, Japan; ^5^ Department of Surgery, Graduate School of Medicine, Kyoto University, Kyoto, Japan; ^6^ Department of Gastroenterology and Hepatology, Graduate School of Medicine, Kyoto University, Kyoto, Japan; ^7^ Department of Radiation Oncology, Japanese Red Cross Society Wakayama Medical Center, Wakayama, Japan

**Keywords:** dynamic tumor tracking (DTT), intensity-modulated radiotherapy (IMRT), chemoradiotherapy, locally advanced pancreatic cancer, prognosis

## Abstract

**Objectives:**

We performed dynamic tumor-tracking IMRT (DTT-IMRT) in locally advanced pancreatic cancer (LAPC) patients using a gimbaled linac of Vero4DRT. The purpose of this study is to report the first clinical results.

**Methods:**

From June 2013 to June 2015, eleven LAPC patients enrolled in this study and DTT-IMRT was successfully performed. The locoregional progression free survival (LRPFS), distant metastasis free survival (DMFS), overall survival (OS), hematologic and gastrointestinal (GI) toxicities were evaluated. Oncologic outcomes were estimated using Kaplan-Meier analysis, and toxicities using CTCAE v4.0.

**Results:**

The median radiation dose was 48 Gy (range, 45-51) in 15 fractions. Concurrent chemoradiotherapy (CCRT) was performed using gemcitabine in 9 patients and S-1 in one, while one patient refused. With a median follow-up of 22.9 months, 1-year LRPFS, DMFS, and OS rates were 90.9%, 70.7%, and 100%, respectively. Median survival time was 23.6 months. Grade-3 leucopenia and neutropenia were observed in two (18%) and one patient (9%), respectively. Grade-2 acute GI toxicity occurred in 2 patients (18%) and late grade-3 in 1 patient (9%).

**Conclusions:**

Preliminarily application of DTT-IMRT using a gimbaled linac on CCRT in LAPC patients resulted in excellent locoregional control and OS without severe toxicity.

## INTRODUCTION

Pancreatic cancer is one of the leading causes of cancer-related mortality [1]. Locally advanced pancreatic cancer (LAPC) remains to be an oncologic challenge, as radical surgical resection is not applicable and the outcomes of chemotherapy with or without radiotherapy are still poor. Unfortunately, radiotherapy for LAPC is often suboptimal. The radiosensitive gastrointestinal (GI) organs limit the radiation dose to the tumors of pancreases. Previous attempts to increase radiation dose using conventional techniques were unsuccessful, resulting in a high morbidity and mortality [2].

Intensity-modulated radiotherapy (IMRT) can simultaneously reduce the dose to surrounding normal organs, while allowing an increase in target tumor dose. IMRT for LAPC is considered useful and is demonstrated to reduce GI toxicities [3]. In addition, several recent reports have demonstrated that dose escalation by IMRT improved local control and overall survival in LAPC [4]. However, because a pancreatic tumor moves mainly due to respiration which can cause discrepancies between planned and actual dose distributions causing unexpected under-dosing of the tumor and/or overdose of the normal tissue, the management of tumor motion is critical for IMRT [5]. There are several ways of motion control including motion-encompassing methods, breath-hold techniques, forced shallow-breathing, respiratory-gated techniques, and dynamic-tumor tracking methods [6]. Of these, the dynamic-tumor tracking method is considered a favorable method due to patient compliance and throughput of the treatment system.

The Vero4DRT (Mitsubishi heavy Industries Ltd., Tokyo, Japan, and BrainLab AG, Feldkirchen, Germany) has two specific features that allow dynamic tumor-tracking IMRT (DTT-IMRT) with real-time monitoring [7]. First, a pair of orthogonal kV X-ray imagers on the gantry can detect in real-time, the three-dimensional tumor position via the pre-implanted fiducial marker. Second is the gimbaled X-ray head, which can swing intensity-modulated beams to the moving tumor. These special features enabled us to perform DTT-IMRT with real-time monitoring for LAPC.

In our institution, we have performed DTT-IMRT for LAPC since 2013. Previously, we evaluated and confirmed the accuracy and reliability of DTT-IMRT [8]. In this study, our aim is to clinically evaluate the effects of DTT-IMRT on outcomes and treatment-related acute and late GI toxicities in LAPC.

## RESULTS

### Patient and tumor characteristics

From June 2013 to June 2015, eleven patients were enrolled in this study. During this period, a total of 21 patients received CCRT for LAPC in our institution. Six patients received three-dimensional conformal radiotherapy (3DCRT) and four received breath-hold IMRT, while the other eleven were enrolled in this study. We did not routinely evaluate the distance of respiratory tumor motion before radiotherapy. Instead, we evaluated it when patients satisfied the eligibility criteria except respiratory tumor motion and wanted to join the study. All the patients we evaluated had greater than 10 mm tumor motion and were enrolled in this study.

All patients received gemcitabine-based induction chemotherapy. For concurrent chemotherapy during radiotherapy, the majority of patients (82%) received gemcitabine, while one patient received S-1 and another patient refused to receive concurrent and maintenance chemotherapy. Gemcitabine administration was completed in six patients, and three patients required one interruption of gemcitabine due to G3 leucopenia, G1 fatigue, or patient's request. Median radiation doses and fractions were 48 Gy in 15 fractions (range, 45-51 Gy), respectively. After chemoradiation, most patients (91%) received maintenance chemotherapy. No patient received curative-intent surgery following DTT-IMRT. Characteristics of the patients, tumors, and treatment are summarized in Table 1.

**Table 1:** Patient characteristics

Characteristic (n = 11)	
Age (median, range)	71, 64 – 79
Gender (male / female)	9 / 2
PS (0 / 1)	5 / 6
Tumor location (head or uncus / body or tail)	5 / 6
Tumor size (median, range[mm])	23, 15 – 40
Clinical Stage (UICC7th) Stage2A/3	1 / 10
Pretreatment CA19-9 (median, range[U/ml])	125, 15 - 1800
Induction CTx (GEM / GEM+S-1 / GEM+nabPTX)	9 / 1 / 1
Concurrent CTx (GEM / S-1 / none)	9 / 1 / 1
Maintenance CTx (GEM / GEM+S-1 / none)	9 / 1 / 1
Radiation dose (median, range[Gy])	48, 45 – 51
Conversion surgery	0

Abbreviations: PS = performance status, UICC = Union for International Cancer Control, CTx = chemotherapy, GEM = gemcitabine, nabPTX = albumin-bound paclitaxel.

### Treatment outcome

**Figure 1:**
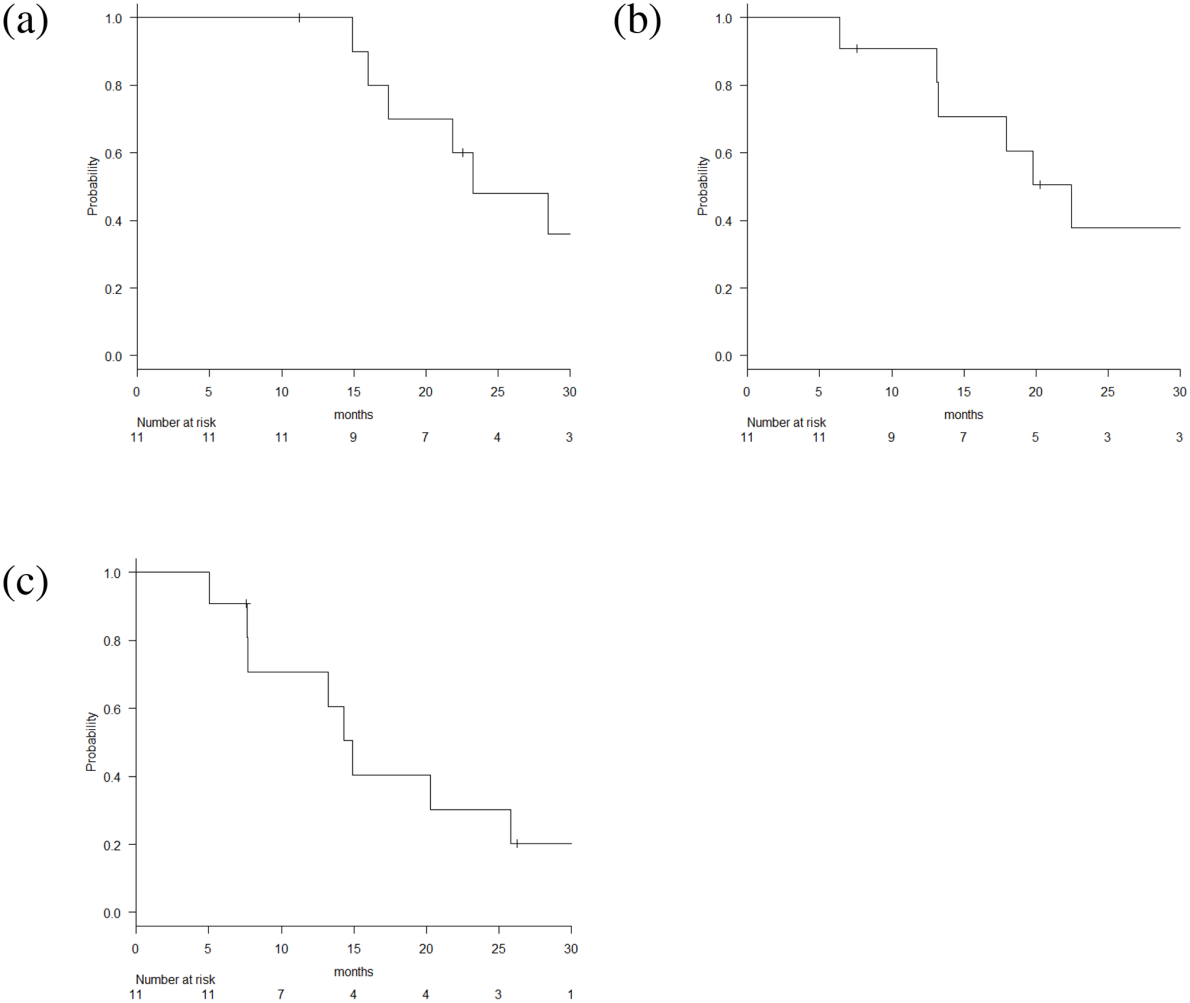
Kaplan-Meier estimates of **(a)** overall survival, **(b)** loco-regional progression free survival and **(c)** distant metastasis free survival.

Median follow-up period was 22.9 months. The 1-year and 2-year OS rates were 100% and 48.0%, respectively. Median survival time (MST) was 23.6 months. The 1-year and 2-year LRPFS rates were 90.9% and 37.9%, respectively. The 1-year and 2-year DMFS rates were 70.7% and 30.3%, respectively (Figure 1). Of the eleven patients, two developed local recurrences, which were the progression of their primary tumors. One patient had local recurrence without distant metastasis 20 months after CRT. The other patient had distant metastases of liver and peritoneum 7.7 months after CRT and had local recurrence at 13.3 months.

Of the eleven patients, seven developed distant metastases. Four of these metastases were in the peritoneum, one was in the liver, one was in an adrenal gland, and one had metastases in both the peritoneum and liver. The chemotherapeutic regimen was gemcitabine for most recurrent cases. The median DMFS was 15.1 months. In the gemcitabine arm of the SCALOP trial, induction chemotherapy consisted of three cycles of gemcitabine (1000 mg/m^2^ on days 1, 8, 15 of a 28 day cycle), followed by radiotherapy in combination with gemcitabine (300 mg/m^2^ once per week, six doses total) [9]. In that trial, the median DMFS of the gemcitabine group was 11.9 months. Compared with our protocol, the regimen of induction chemotherapy was the same, but the dose of concurrent chemotherapy was different. We used full-dose gemcitabine (1000 mg/m^2^) concurrently with radiotherapy. We thought that our treatment has an advantage by enabling full-dose concurrent chemotherapy, which may lead to better DMFS. After recurrence, we changed the regimen of chemotherapy from gemcitabine to an S-1-based regimen in most cases.

### Toxicities

An overview of acute hematologic toxicities and their grades is shown in Table 2. An overview of the acute and late GI toxicities and their grades is shown in Table 3. Acute GI toxicity of grade 2 occurred in 2 patients (18%). Late GI toxicity of grade 3 occurred in 1 patient (9%), who developed G3 anemia during the maintenance chemotherapy after DTT-IMRT without any disease progression. GI bleeding was suspected but ruled out upon upper and total colon endoscopy. The patient stopped chemotherapy, received several blood transfusions, and was followed-up thereafter. Repeated upper endoscopy revealed GI bleeding as gastric antral vascular ectasia at 20 months after CRT, and the patient received argon plasma coagulation treatment, which improved the anemia. The patient did not resume chemotherapy because of no evidence of disease progression at the time, and for over 3 years from induction chemotherapy. GI toxicity of grade 4 or higher was not observed.

**Table 2:** Acute hematologic toxicity

	Gr 0-1	Gr 2	Gr 3	Gr 4-5
Leucopenia	4 (36%)	5 (46%)	2 (18%)	0 (0%)
Neutropenia	8 (73%)	2 (18%)	1 (9%)	0 (0%)
Anemia	9 (92%)	2 (18%)	0 (0%)	0 (0%)
Thrombocytopenia	10 (91%)	1 (9%)	0 (0%)	0 (0%)

Abbreviation: Gr = grade.

**Table 3:** Acute and late gastrointestinal toxicity

	Gr 0-1	Gr 2	Gr 3	Gr 4-5
Acute GI toxicity	9 (82%)	2 (18%)	0 (0%)	0 (0%)
Late GI toxicity	10 (91%)	0 (0%)	1 (9%)	0 (0%)

Abbreviations: GI = gastrointestinal, Gr = grade.

## DISCUSSION

This is the first study to report the clinical results of LAPC patients treated with DTT-IMRT with real-time monitoring using a gimbal mounted linac.

DTT-IMRT is a technique that we developed and realized for the first time in the world. We have performed DTT-stereotactic body radiotherapy (SBRT) for lung tumors since September 2011 and liver tumors since March 2013 using the Vero4DRT system [10, 11]. Our previous study on DTT-SBRT for lung and liver tumors demonstrated that planning target volume (PTV) size was significantly decreased and the dose to OAR was significantly reduced. In addition, the tumor tracking accuracy was high. We started performing DTT-IMRT for LAPC patients in June 2013. We evaluated and confirmed the accuracy and reliability of DTT-IMRT [8].

In this study, we evaluated the clinical results of patients treated with DTT-IMRT in a sufficient follow-up period of 22.9 months. The profiles of clinical outcome and toxicities were promising. The MST and 1-year LRPFS were 23.6 months and 90.9%, and severe late GI toxicity was observed in only one patient. Shibuya et al. have reported the results of a phase II study using radiation therapy combined with weekly low-dose gemcitabine for LAPC [12]. In the trial, the radiation technique was 3DCRT, and radiation dose was 54 Gy in 30 fractions and gemcitabine was administered weekly at 250 mg/m^2^. The 1-year survival rate was 74% and MST was 16.6 months. Of 21 patients, six (29%) had local relapse as initial disease progression. Grade-3 gastric ulcers in 10% of patients, but no grade 4 or 5 toxicities were observed. In the recently published LAP07 randomized clinical trial, the radiation technique was three-dimensional conformal radiotherapy (3DCRT), prescribed dose was 54 Gy in 30 fractions and capecitabine was given at a dose of 800 mg/m^2^ twice daily on the days of radiation therapy. The MST of CRT group was 15.2 months and Grade 3 or 4 adverse events were observed in 23.1% of patients [13]. Compared to the results of our previous 3DCRT trial and the LAP07 trial, the results of DTT-IMRT are favorable with respect to efficacy and toxicity.

At our institution, we have performed IMRT for LAPC since 2009. For introduction of IMRT in our institution, we adopted the breath-hold method for motion management [14]. However, it is sometimes uncomfortable for patients to hold their breath during irradiation and there are uncertainties regarding reproducibility of the repeated breath-hold position [15]. In addition, as the rate of distant metastasis in LAPC patients is high, it is important that radiotherapy can be performed without decreasing the intensity of chemotherapy [12]. In our protocol, we were able to use a standard dose of chemotherapy concurrently. However, standard dose chemotherapy tends to increase the rate and degree of adverse events such as fatigue and nausea/vomiting. In this setting, respiratory training or prolonged treatment time with radiation is a significant burden for patients. To solve these problems, we used Vero4DRT to enable dynamic tumor-tracking with real time monitoring. Pancreatic tumor moves considerably both inter- and intrafractional, dynamic tumor-tracking is appropriate for management of respiratory motion [5]. Dynamic tumor-tracking is also favorable because respiratory training is not needed and the treatment time is not prolonged. It is clinically beneficial both for patient comfort and the throughput of treatment system. This low burden of the procedure allows treatment of larger populations such as elderly patients.

The disadvantages of DTT-IMRT using the Vero4DRT system requires the inserting a fiducial marker before radiotherapy. For DTT-IMRT, the fiducial gold marker needs to be implanted inside or near the tumor percutaneously or endoscopically as an internal surrogate marker for the tumor position. The fiducial marker is useful not only for DTT-IMRT but also for image guided radiation therapy (IGRT). Without the fiducial marker, it is quite difficult to distinguish the pancreatic tumor from surrounding normal organs such as stomach and duodenum on cone beam CT for IGRT because of the movement or gas from gastrointestinal organs. Several reports demonstrated that marker matching is better than bony structure matching for pancreatic cancer [16, 17]. Considering that the fiducial marker is beneficial to be implanted for IGRT for pancreatic radiotherapy if it is not DTT-IMRT, indispensability of fiducial marker for DTT-IMRT is not a specific weak point.

There are several limitations in this study. First, this was a single institution study with a small number of patients. Because the DTT-IMRT technique was realized for the first time at our institution, we evaluated it here first, allowing future multicenter trials. Second, there is a possibility of selection bias in enrolling patients. During this period, we treated LAPC patients not only using DTT-IMRT but also breath-hold IMRT or 3DCRT. However, we recruited all types of LAPC patients if they satisfied the conditions and agreed with the trial. All clinical decisions were made by a multidisciplinary tumor board, a process that reduced selection bias.

In conclusion, DTT-IMRT with real-time monitoring using a gimbal mounted linac was clinically feasible for LAPC with low toxicity. To prospectively evaluate this protocol in a multicenter setting, we are currently conducting a phase II multi-institutional clinical trial of DTT-IMRT in LAPC patients (UMIN000017521).

## MATERIALS AND METHODS

### Patients

Eligibility criteria for this study were as follows: (1) patients with clinical stage II-III unresectable LAPC, (2) patients did not receive curative-intent surgery or radiotherapy, (3) pathological confirmation of adenocarcinoma, (4) distance of respiratory tumor motion was over 10 mm, (5) written informed consent. Locally advanced unresectable disease was defined as superior mesenteric artery or celiac axis encasement >180 degrees, unreconstructable superior mesenteric vein/portal occlusion, or aortic invasion without distant metastasis. Resectability was determined by a multidisciplinary panel of surgeons, radiologists, and medical and radiation oncologists. This was a single-institution study, approved by our Institutional Review Board.

### Pre-planning procedures

Prior to treatment planning, a gold marker (0.5 or 0.75 ×10 mm, Visicoil, IBA dosimetry, Louvain-la-neuve, Belgium) was implanted inside or near the tumor percutaneously or endoscopically as an internal surrogate marker for the tumor position. A gold marker was implanted percutaneously when the patients received exploratory laparoscopy with suspicion of peritoneal dissemination, and the pathological diagnosis was negative. Otherwise, a marker was implanted endoscopically using a 22-gauge needle. Initially, each marker was planned to be implanted into the tumor. However, if it was difficult to put the marker into the tumor because of adjacent vessels, it was instead implanted near the tumor. Each marker was implanted straight, rather than bent, to be easily detected by X-ray imagers during treatment. Treatment planning was performed at least 1 week after insertion of the gold marker.

After at least 3 hours of fasting, a CT simulation was performed. The patient was fixed in the supine position with both arms raised using BodyFIX system (Elekta, Stockholm, Sweden). The patient was examined by end-expiratory breath-hold contrast-enhanced CT and subsequent four-dimensional CT (4D-CT) with free-breathing using the LightSpeedRT 16-slice CT simulator (GE Healthcare, Little Chaulfont, United Kingdom) and a real-time positioning management system (Varian Medical System, Palo Alto, CA). The periodic whole images were sorted into 10 phased bins of 4D-CT images using the Advantage Workstation (GE Healthcare). The breath-hold contrast-enhanced CT was used as a reference for treatment planning. Following CT simulation, the patient was transferred to the Vero4DRT to perform 4D modeling, which correlated the external abdominal motion and internal fiducial gold maker motion to assess the 4D modeling error [5, 10].

### Treatment planning

The gross tumor volume (GTV) included the pancreatic tumor and metastatic lymph nodes. The clinical target volume (CTV) was defined as GTV plus a 5-mm margin in all directions, in addition to the retropancreatic space and para-aortic lymph nodes between the celiac axis and the superior mesenteric artery. The GTVs, CTVs, stomach, and duodenum were delineated on all 10 phases of 4D-CT and were overlaid onto a mid-ventilation phase. PTV margin was defined as the margin for setup error in addition to the margins for the 4D modelling error, the baseline drift of abdominal position, and mechanical errors, with a minimum of 5 mm. The PTV was generated by adding at least a 5-mm margin to the CTV. IMRT planning was performed using iPlan RT Dose (BrainLab). The prescription dose was specified as D95 (the dose that covers 95% of the structure) to PTV-boost, a volume that subtracted normal organs (the stomach and the duodenum) plus 3 to 7-mm margins from PTV, depending on the proximity of the normal organs to the tumor. The prescription dose was individualized between 45 and 51 Gy in 15 fractions by achieving the dose constraint for organ-at-risk (OAR) and referring to a previous institutional trial (UMIN000004589). The dose constraints are shown in Table 4. The standard beam arrangement involved six gantry angles.

**Table 4:** Dose constraints for organs at risk

Structure	Constraints
Stomach	V45Gy<1 cc
	V42Gy<5 cc
	V39Gy<25 cc
Duodenum	V45Gy<1 cc
	V42Gy<5 cc
	V39Gy<25 cc
Stomach+PRV	V39Gy<30 cc
	V36Gy<45 cc
Duodenum+PRV	V39Gy<30 cc
	V36Gy<45 cc
Spinal cord	Dmax<36 Gy
Spinal cord+PRV	D2cc<39 Gy
Kidney	V20Gy<30%
Liver	Dmean<30 Gy

Abbreviations: PRV = planning organ at risk volume, Dmax = the maximum dose to the structure volume, Dmean = the mean dose to the structure volume, D2cc = the maximum dose covering ≥2cc of the structure volume, VxxGy = the volume of the structure receiving > xxGy.

### Irradiation of treatment beams

The patient was placed in BodyFix and initial set-up error was corrected based on bony structures. Next, a 4D model was built to correlate the infrared markers on abdomen with the internal gold fiducial marker [8]. The gimbaled X-ray head of Vero4DRT could swing the beams to the target predicted by the 4D model based on the infrared marker on the patient's abdominal wall under free-breathing. During irradiation, the position of the fiducial marker was monitored visually using the kV X-ray and MV X-ray imagers every second. The predicted marker position was overlaid on the kV images. If the fiducial marker was displaced from the predicted position by 3 mm frequently, the irradiation was interrupted and rebuilding the 4D model was considered [10, 11].

### Chemotherapy

The regimen of induction chemotherapy consisted of weekly intravenous administration of 1000 mg/m^2^ gemcitabine for 4-weeks. The regimens of chemoradiotherapy (CRT) consisted of weekly gemcitabine 1000 mg/m^2^. As additional treatment after radiotherapy, 3 weekly doses of gemcitabine at 1000 mg/m^2^ every 28 days were administered until the tumor progressed or patient refused. If patients had complications associated with gemcitabine, such as intestinal pneumonia, 80 mg/m^2^/day of S-1 was administered orally during radiotherapy twice daily on weekdays.

### Follow-up after treatment

Patients were followed-up every 1.5-2 months after the completion of radiotherapy. History and physical examination, a complete blood count, serum chemistry, and tumor makers such as CA19-9 were obtained on every follow-up visit. CT scans or PET-CT scans were obtained every 3-4 months. All toxicities were scored according to the Common Terminology Criteria for Adverse Events (CTCAE), version 4.0.

### Statistics

Overall survival (OS) was defined as the period from chemotherapy start date to the date of death due to any cause, and it was censored at the last follow-up visit for living patients. The Kaplan-Meier method was used to estimate the OS, locoregional progression free survival (LRPFS), distant metastasis free survival (DMFS). All statistical analyses were performed using EZR version 1.11 (Saitama Medical Center, Jichi Medical University, Saitama, Japan), which is a graphical user interface for R version 2.13.2 (The R Foundation for Statistical Computing, Vienna, Austria).

## Abbreviations

DTT: dynamic tumor-tracking
IMRT: intensity-modulated radiotherapy
LAPC: locally advanced pancreatic cancer
LRPFS: locoregional progression free survival
DMFS: distant metastasis free survival
OS: overall survival
GI: gastrointestinal
CCRT: concurrent chemoradiotherapy
MST: median survival time
SBRT: stereotactic body radiotherapy
PTV: planning target volume
3DCRT: three-dimensional conformal radiotherapy
IGRT: image guided radiation therapy
4D-CT: four-dimensional CT
GTV: gross tumor volume
CTV: clinical target volume
CTCAE: Common Terminology Criteria for Adverse Events

## References

[1] Hidalgo M (2010). Pancreatic cancer. N Engl J Med.

[2] Chauffert B, Mornex F, Bonnetain F, Rougier P, Mariette C, Bouche O, Bosset JF, Aparicio T, Mineur L, Azzedine A, Hammel P, Butel J, Stremsdoerfer N (2008). Phase III trial comparing intensive induction chemoradiotherapy (60 Gy, infusional 5-FU and intermittent cisplatin) followed by maintenance gemcitabine with gemcitabine alone for locally advanced unresectable pancreatic cancer. Definitive results of the 2000-01 FFCD/SFRO study. Ann Oncol.

[3] Bittner MI, Grosu AL, Brunner TB (2015). Comparison of toxicity after IMRT and 3D-conformal radiotherapy for patients with pancreatic cancer - a systematic review. Radiother Oncol.

[4] Krishnan S, Chadha AS, Suh Y, Chen HC, Rao A, Das P, Minsky BD, Mahmood U, Delclos ME, Sawakuchi GO, Beddar S, Katz MH, Fleming JB (2016). Focal Radiation Therapy Dose Escalation Improves Overall Survival in Locally Advanced Pancreatic Cancer Patients Receiving Induction Chemotherapy and Consolidative Chemoradiation. Int J Radiat Oncol Biol Phys.

[5] Akimoto M, Nakamura M, Nakamura A, Mukumoto N, Kishi T, Goto Y, Mizowaki T, Hiraoka M (2017). Inter- and Intrafractional Variation in the 3-Dimensional Positions of Pancreatic Tumors Due to Respiration Under Real-Time Monitoring. Int J Radiat Oncol Biol Phys.

[6] Keall PJ, Mageras GS, Balter JM, Emery RS, Forster KM, Jiang SB, Kapatoes JM, Low DA, Murphy MJ, Murray BR, Ramsey CR, Van Herk MB, Vedam SS (2006). The management of respiratory motion in radiation oncology report of AAPM Task Group 76. Med Phys.

[7] Kamino Y, Takayama K, Kokubo M, Narita Y, Hirai E, Kawawda N, Mizowaki T, Nagata Y, Nishidai T, Hiraoka M (2006). Development of a four-dimensional image-guided radiotherapy system with a gimbaled X-ray head. Int J Radiat Oncol Biol Phys.

[8] Mukumoto N, Nakamura M, Yamada M, Takahashi K, Akimoto M, Miyabe Y, Yokota K, Kaneko S, Nakamura A, Itasaka S, Matsuo Y, Mizowaki T, Kokubo M (2016). Development of a four-axis moving phantom for patient-specific QA of surrogate signal-based tracking IMRT. Med Phys.

[9] Mukherjee S, Hurt CN, Bridgewater J, Falk S, Cummins S, Wasan H, Crosby T, Jephcott C, Roy R, Radhakrishna G, McDonald A, Ray R, Joseph G (2013). Gemcitabine-based or capecitabine-based chemoradiotherapy for locally advanced pancreatic cancer (SCALOP): a multicentre, randomised, phase 2 trial. Lancet Oncol.

[10] Matsuo Y, Ueki N, Takayama K, Nakamura M, Miyabe Y, Ishihara Y, Mukumoto N, Yano S, Tanabe H, Kaneko S, Mizowaki T, Monzen H, Sawada A (2014). Evaluation of dynamic tumour tracking radiotherapy with real-time monitoring for lung tumours using a gimbal mounted linac. Radiother Oncol.

[11] Iizuka Y, Matsuo Y, Ishihara Y, Akimoto M, Tanabe H, Takayama K, Ueki N, Yokota K, Mizowaki T, Kokubo M, Hiraoka M (2015). Dynamic tumor-tracking radiotherapy with real-time monitoring for liver tumors using a gimbal mounted linac. Radiother Oncol.

[12] Shibuya K, Oya N, Fujii T, Doi R, Nakamura A, Matsuo Y, Mitsumori M, Hiraoka M (2011). Phase II study of radiation therapy combined with weekly low-dose gemcitabine for locally advanced, unresectable pancreatic cancer. Am J Clin Oncol.

[13] Hammel P, Huguet F, van Laethem JL, Goldstein D, Glimelius B, Artru P, Borbath I, Bouche O, Shannon J, Andre T, Mineur L, Chibaudel B, Bonnetain F (2016). Effect of Chemoradiotherapy vs Chemotherapy on Survival in Patients With Locally Advanced Pancreatic Cancer Controlled After 4 Months of Gemcitabine With or Without Erlotinib: The LAP07 Randomized Clinical Trial. JAMA.

[14] Nakamura M, Shibuya K, Shiinoki T, Matsuo Y, Nakamura A, Nakata M, Sawada A, Mizowaki T, Hiraoka M (2011). Positional reproducibility of pancreatic tumors under end-exhalation breath-hold conditions using a visual feedback technique. Int J Radiat Oncol Biol Phys.

[15] Nakamura M, Shibuya K, Nakamura A, Shiinoki T, Matsuo Y, Nakata M, Sawada A, Mizowaki T, Hiraoka M (2012). Interfractional dose variations in intensity-modulated radiotherapy with breath-hold for pancreatic cancer. Int J Radiat Oncol Biol Phys.

[16] Yu S, Lawrenson L, Wei R, Sehgal V, Hanna N, Kuo J, Daroui P, Ramsinghani N, Al-Ghazi M (2016). The dosimetric impact of image guided radiation therapy by intratumoral fiducial markers. Pract Radiat Oncol.

[17] Packard M, Gayou O, Gurram K, Weiss B, Thakkar S, Kirichenko A (2015). Use of implanted gold fiducial markers with MV-CBCT image-guided IMRT for pancreatic tumours. J Med Imaging Radiat Oncol.

